# Supplementary material for: Follicle architecture and innervation of functionally distinct rat vibrissae
Source: Commun Biol. 2025 Jul 1;8:979. doi: 10.1038/s42003-025-08336-w (PMC12216610; doi:10.1038/s42003-025-08336-w)
Supplement: Supplementary file 2 — Supplementary Material [file 42003_2025_8336_MOESM2_ESM.pdf]

Supplementary Table

Table S1 Description of staining and origin of the analyzed vibrissa follicles.

**Sample**	**Animal**	**Staining**	**Embedding**	**Analysis**
Trident 1	240904_1	1% OsO4	Eppon	Full reconstruction
Trident 2	230606_1	2% OsO4, 4% UA	Paraffin	Full reconstruction
Trident 3	240828_1	1% OsO4	Eppon	Axon count
Micro 1	230606_1, left	2% OsO4, 4% UA	Paraffin	Full reconstruction
Micro 2	230606_1, right	2% OsO4, 4% UA	Paraffin	Full reconstruction
Micro 3	240904_1, left	1% OsO4	Eppon	Axon count
lSO 1	230606_1, left	2% OsO4, 4% UA	Paraffin	Full reconstruction
lSO 2	240828_1, right	1% OsO4	Eppon	Axon count
lSO 3	240903_1, left	1% OsO4	Eppon	Axon count
C2 1	231017_1, left	1% OsO4	Paraffin	Full reconstruction
C2 2	230606_1, right	2% OsO4, 4% UA	Paraffin	Full reconstruction
C2 3	230602_1, left	None	Paraffin	Full reconstruction

Supplementary Figure

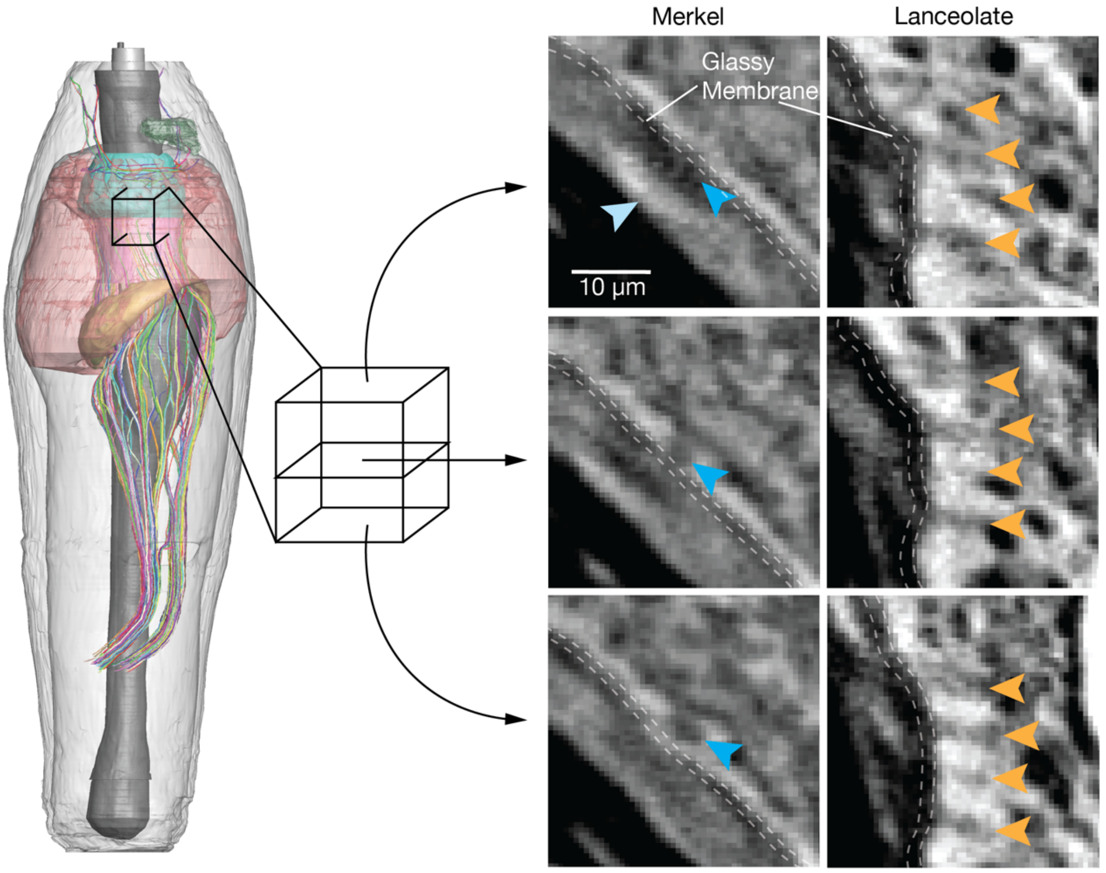

Fig. S1 Fine details of Merkel and lanceolate endings.

Left: 3d rendering of a C2 vibrissa follicle. Box indicates the position of the subvolume from which sections are shown on the right.

Center: 2d sections of a z-stack showing the piercing of merkel afferents through the glassy membrane before making contact with a merkel cell. Blue arrows = merkel afferent, light blue arrow = merkel cell.

Right: 2d sections of a z-stack showing the attachment of lanceolate endings to the glassy membrane.
