# Supplementary material for: Follicle architecture and innervation of functionally distinct rat vibrissae
Source: Commun Biol. 2025 Jul 1;8:979. doi: 10.1038/s42003-025-08336-w (PMC12216610; doi:10.1038/s42003-025-08336-w)
Supplement: Supplementary file 3 — Description of Additional Supplementary Files [file 42003_2025_8336_MOESM3_ESM.pdf]

Description of Additional Supplementary files

File name: Supplementary Data 1

Supplementary Data 1 contains the source data for all figures
